# Physical and Biological Properties of a Chitosan Hydrogel Scaffold Associated to Photobiomodulation Therapy for Dental Pulp Regeneration: An *In Vitro* and *In Vivo* Study

**DOI:** 10.1155/2021/6684667

**Published:** 2021-01-25

**Authors:** Maria Stella Moreira, Giovanna Sarra, Giovanna Lopes Carvalho, Flavia Gonçalves, Hector Valentin Caballero-Flores, Ana Clara Fagundes Pedroni, Cesar Angelo Lascala, Luiz Henrique Catalani, Márcia Martins Marques

**Affiliations:** ^1^Post-Graduation Program in Dentistry, School of Dentistry, Ibirapuera University, São Paulo, Brazil; ^2^A.C. Camargo Cancer Center, Stomatology Department, São Paulo, Brazil; ^3^Department of Restorative Dentistry, School of Dentistry, Universidade de São Paulo, São Paulo, Brazil; ^4^Department of Stomatology, School of Dentistry, Universidade de São Paulo, São Paulo, Brazil; ^5^Department of Fundamental Chemistry, Chemical Institute, Universidade de São Paulo, São Paulo, Brazil

## Abstract

**Background:**

The regeneration of dental pulp, especially in cases of pulp death of immature teeth, is the goal of the regenerative endodontic procedures (REPs) that are based on tissue engineering principles, consisting of stem cells, growth factors, and scaffolds. Photobiomodulation therapy (PBMT) showed to improve dental pulp regeneration through cell homing approaches in preclinical studies and has been proposed as the fourth element of tissue engineering. However, when a blood clot was used as a scaffold in one of these previous studies, only 30% of success was achieved. The authors pointed **out** the instability of the blood clot as the regeneration shortcoming. Then, to circumvent this problem, a new scaffold was developed to be applied with the blood clot. The hypothesis of the present study was that an experimental injectable chitosan hydrogel would facilitate the three-dimensional spatial organization of endogenous stem cells in dental pulp regeneration with no interference on the positive influence of PBMT.

**Methods:**

For the *in vitro* analysis, stem cells from the apical papilla (SCAPs) were characterized by flow cytometry and applied in the chitosan scaffold for evaluating adhesion, migration, and proliferation. For the *in vivo* analysis, the chitosan scaffold was applied in a rodent orthotopic dental pulp regeneration model under the influence of PBMT (660 nm; power output of 20 mW, beam area of 0.028 cm^2^, and energy density of 5 J/cm^2^).

**Results:**

The scaffold tested in this study allowed significantly higher viability, proliferation, and migration of SCAPs *in vitro* when PBMT was applied, especially with the energy density of 5 J/cm^2^. These results were in consonance to those of the *in vivo* data, where pulp-like tissue formation was observed inside the root canal.

**Conclusion:**

Chitosan hydrogel when applied with a blood clot and PBMT could in the future improve previous results of dental pulp regeneration through cell homing approaches.

## 1. Introduction

Dental pulp is a connective tissue with neuroectodermic origin that is confined inside rigid dentine walls. The blood vessels of dental pulp enter and leave the tooth only through the apical foramen. In case of dental pulp necrosis in response to harmful stimuli, such as caries or trauma, this anatomical situation impairs dental pulp regeneration. The regeneration of dental pulp, especially in cases of pulp death of immature teeth, is the goal of the regenerative endodontic procedures (REPs) that are based on tissue engineering elements, consisting of stem cells, growth factors, and scaffolds [1–3]. Recent advances in dental pulp tissue engineering allow for a gradual translation from bench to clinic. In particular, tissue engineering based on cell homing approaches in revitalization procedures offers a biology-based and clinically feasible treatment option for teeth with pulp necrosis.

Photobiomodulation therapy (PBMT) was proposed as another important element for tissue engineering because PBMT has properties able to improve stem cell response [4]. PBMT when applied with adequate parameters improves the *in vitro* stem cell proliferation, migration, and differentiation [4–9]. Additionally, in preclinical and clinical studies, the PBMT has shown improvement in the regeneration of tissues surrounded by mineralized tissues, where the vascularization can be compromised, such as the jaw bone [10, 11] and dental pulp [12–14].

Moreira et al. showed that the pulp revitalization procedures based on cell homing approaches evoked a blood clot into the root canal as a scaffold for dental pulp regeneration. The authors showed success only when applying PBMT. However, the authors pointed out the instability of the blood clot as a regeneration shortcoming. This showed the importance of the scaffold quality to enhance the scaffolds' cellular activities of stem cells for the success of dental pulp regeneration.

Advances in the development of scaffold materials have made biological therapies in regenerative endodontic treatment procedures more effective and feasible. The hydrogels from natural origin have been frequently used as scaffolds because they are composed of molecules of the extracellular matrix (ECM) or of components similar to ECM molecules [15, 16]. Gelatin, collagen, hyaluronic acid, alginate, and chitosan are some examples of natural scaffolds [16]. However, the most appropriate injectable natural scaffold for dentin pulp regeneration has not been developed [16, 17]. Among the range of existing scaffolds, chitosan is a widely used biomaterial, which has several biological properties [17–19].

Chitosan is a bioactive natural polysaccharide that has great biocompatibility, biodegradability, and hemostatic potential; it promotes cell adhesion, proliferation, and differentiation; it has a broad antimicrobial effect; it does not cause an immune reaction; it is not carcinogenic; and in addition, having a potential for cell homing has also been associated to this biomaterial [20–27]. Furthermore, chitosan can be used as a thermosensitive hydrogel whose gelation occurs above 37°C [28]; this characteristic allows the injection of the material in a fluid state inside the pulp chamber and gelation of the biomaterial by the body temperature, increasing the stability of the biomaterial and being a key factor for applications in pulp regeneration. Aimed at presenting an alternative tissue engineering approach for regenerating tissues surrounded by mineralized tissue, such as the dental pulp, the objective of this study was to evaluate the biological responses of stem cells grown inside an experimental injectable hydrogel chitosan scaffold and submitted to PBMT and evaluate the pulp regeneration capacity in an orthotopic model in rats. The hypothesis of this study was that an experimental injectable chitosan hydrogel would facilitate the three-dimensional spatial organization of endogenous stem cells in dental pulp regeneration associated with PBMT on these cells.

## 2. Materials and Methods

The study was approved by the Ethical Committee of the School of Dentistry of the University of São Paulo (#CAAE 40392214.5.0000.0075).

### 2.1. Biomaterial

#### 2.1.1. Chitosan Synthesis

The high-molecular weight and low-acetylation chitosan powder was dissolved in 0.1 M hydrochloric acid solution (Synth, SP, Brazil). After complete solubilization, the chitosan solution (11 mg/l concentration; pH 7.4) was kept under stirring on an ice bath. Then, a solution of *β*-glycerophosphate disodium salt hydrate (crosslinker) in the concentration of 286 mg/ml (20% *w*/*v*) was slowly dripped. After homogenization, this pregel solution was stored at 8°C. The experiments were conducted keeping the biomaterial constantly refrigerated in ice chips.

#### 2.1.2. Chitosan Viscosity

Chitosan pregel when incubated at 37°C for 1 hour becomes a hydrogel. The initial viscosity of this chitosan hydrogel was measured. Then, the chitosan hydrogel was kept at 37°C at two different conditions: in the presence of phosphate-buffered saline (PBS chitosan hydrogel (CHP) group) or in the absence of PBS (PBS absent chitosan hydrogel (CHPA) group). In the CHP group, the PBS was added two hours after the incorporation of the crosslinker into the chitosan solution (2 ml of hydrogel/1 ml of PBS). The viscosity was measured at 24, 72, or 168 hours in a Brookfield viscometer (Model DV-III) with spindle 52 at 40°C and 250 rpm, 5 minutes after rotation started.

#### 2.1.3. Scanning Electron Microscopy (SEM)

Hydrogel ultrastructure was observed in SEM images. Specimens of chitosan hydrogel were frozen at -20°C for 24 h to further obtain mechanically fractured surfaces. Fractured samples were then placed on metallic stubs, gold-sputtered, and evaluated in scanning electron microscopy.

#### 2.1.4. Stem Cell Culture and Flow Cytometry Analysis

Human SCAPs (stem cells from the apical papilla) isolated from adult teeth were grown in clonogenic medium: *α*-Minimum Essential Medium complemented with 15% fetal bovine serum (FBS), 100 *μ*M L-ascorbic acid-2-phosphate, 100 *μ*g/ml streptomycin, 100 U/ml penicillin, and 2 mM L-glutamine (all from Gibco/Invitrogen, Grand Island, NY, US). Aliquots of SCAPs (1 × 10^6^ cells) were used for analyzing their immunophenotype profile, as previously described [29].

#### 2.1.5. Three-Dimensional Cell Culture: Cell Adhesion Assay

A volume of 1 ml of chitosan pregel (chitosan solution+crosslinker) was placed at the bottom of a 35 mm diameter culture plate. The mixture in the plates was homogenized in a shaker and incubated in a humid atmosphere containing 5% CO_2_, at 37°C, for 10 minutes to allow hydrogel formation. Then, SCAPs (1 × 10^6^ cells/plate) were plated on the top of the chitosan hydrogel. These SCAPs were analyzed under SEM 24 hours after seeding, following the same SEM methodology described above.

#### 2.1.6. Three-Dimensional Cell Culture: Proliferation Assay

For cell proliferation assay, 100 *μ*l of chitosan solution was placed at the bottom of 96-well plates. SCAP pellets were resuspended in the crosslinker (1 × 10^5^ cells per well) and then incorporated into the chitosan solution inside the wells. The mixture in the plates was homogenized in a shaker. This pregel (chitosan solution+crosslinker) containing the cells was then incubated in a humid atmosphere with 5% CO_2_ at 37°C for 10 minutes to allow hydrogel formation. The plates were assigned to one of the following three experimental groups: Group 1 (positive control): no further treatment (nonirradiated); Group 2 (PBMT 3 J/cm^2^): cells treated with PBMT using 3 J/cm^2^; and Group 3 (PBMT 5 J/cm^2^): cells treated with PBMT using 5 J/cm^2^.

The cell viability of SCAPs grown inside the chitosan hydrogel under PBMT (3 or 5 J/cm^2^) or not (control) was analyzed using the MTT reduction assay, as described elsewhere [30] at 72 and 120 hours after seeding. The absorbance was measured in a microplate reader (Biotek II, Biochrom Ltd., Eugendorf, Austria) with a 562 nm filter.

#### 2.1.7. Photobiomodulation Therapy

Photobiomodulation therapy was applied using a continuous-wave indium-gallium-aluminum-phosphide (InGaAlP) diode laser (660 nm; DMC, São Carlos, SP, Brazil) with a spot size of 0.028cm^2^. The following parameters were used in contact and punctual irradiation mode: 20 mW, 0.71 W/cm^2^, 3 J/cm^2^ (4 s) or 5 J/cm^2^ (7 s), and 0.08 J or 0.14 J, respectively. Irradiations were applied twice (6 and 12 h after seeding) underneath each well (for 96-well plates in the center and for 24-well plates in five equidistant points). The output power was checked with a power meter (LaserCheck, Coherent Inc., Santa Clara, CA, US) before and after the irradiations. The control groups were under the same conditions as the irradiated groups, but the laser equipment was kept off. The PBMT parameters were defined as previously described in the literature [5, 7, 14].

#### 2.1.8. Three-Dimensional Cell Culture: Migration Assay

**Figure 1:**
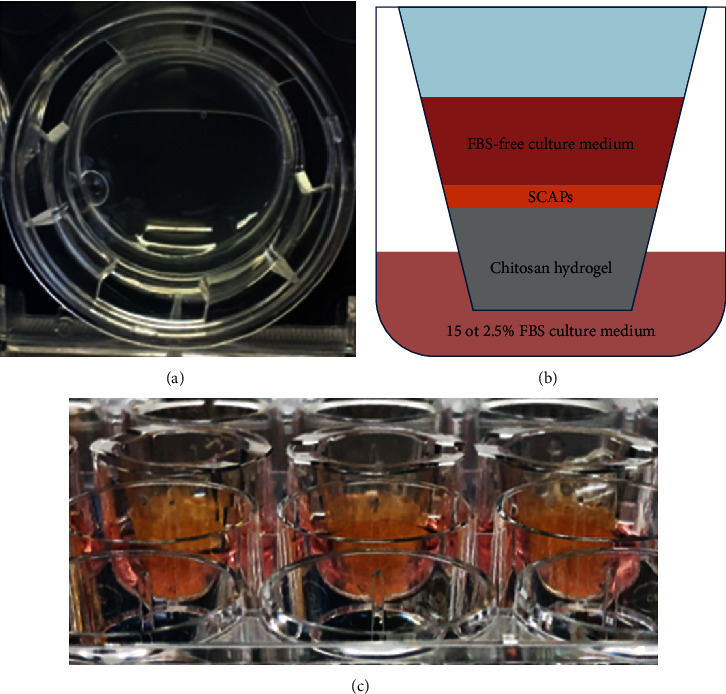
(a) Aspect of the chitosan hydrogel in the insert. (b) Diagram showing a Transwell insert (upper chamber) placed in the culture well (lower chamber) separated by an 8 *μ*m pore size filter. The lower chamber contains culture medium supplemented with 15 or 2.5% of FBS, whereas the upper chamber presents serum-free culture medium. (c) System for Transwell migration assay.

Transwell migration assay was carried out with a Transwell system (Corning, New York, NY, US) composed of a Transwell insert (upper chamber) placed in the culture wells (lower chamber) of 24-well plates separated by an 8 *μ*m pore size filter. A volume of 500 *μ*l of chitosan pregel was placed at the upper chamber on the top of the filter. The plates were maintained on shaker agitation and incubation as previously described, allowing hydrogel formation (Figure 1(a)). Figures 1(b) and 1(c) illustrate the preparation of the migration assay and the system. Culture medium supplemented with 15% FBS (positive control) or 2.5% FBS (negative control and both PBMT groups) was added at the lower chamber. The cells were seeded at the top of the chitosan hydrogel (1 × 10^5^ cells per insert), and the upper chamber was filled with 200 *μ*l of serum-free culture medium. Then, these Transwell plates were assigned to one of the four experimental groups: Group 1 (positive control): lower chamber containing culture medium complemented with 15% FBS; Group 2 (negative control): lower chamber containing culture medium complemented with 2.5% FBS; Group 3 (PBMT 3 J/cm^2^): lower chamber containing culture medium complemented with 2.5% FBS and treated with PBMT using 3 J/cm^2^; and Group 4 (PBMT 5 J/cm^2^): lower chamber containing culture medium complemented with 2.5% FBS and treated with PBMT using 5 J/cm^2^. The cell migration was analyzed in all experimental groups at 18 and 48 hours after the SCAP seeding as previously described [31]. Then, the absorbance was measured with a microplate reader (Biotek II, Biochrom Ltd., Eugendorf, Austria) with a 560 nm filter.

#### 2.1.9. *In Vivo* Orthotopic Dental Pulp Model

Thirty-four adult male Wistar rats (*Rattus norvegicus albinus*) weighing an average of 350 to 375 g and approximately 13 weeks old were used in this study. The rats were anesthetized and fixed on a frame/operative board. Dental pulp from the upper right first molar was surgically exposed, and the pulp of the mesial root canal was removed through instrumentation up to ISO 25. Then, the animals were randomly divided into 4 groups:

- Chitosan scaffold
- Chitosan scaffold+PBMT
- Blood clot+chitosan scaffold
- Blood clot+chitosan scaffold+PBMT

After canal root preparation, a blood clot was evoked as described previously for the hybrid scaffold groups [14], and the root canal was filled with 5 *μ*l of chitosan. The PBMT was carried out in contact with the crown of the tooth, over the mix of blood and chitosan, in a single point, as previously described [14]. A red laser was chosen because of its interaction with the blood clotting hemoglobin. The animals in the groups without PBMT were kept in the same condition as animals of the PBMT groups, but with the laser equipment turned off. Rats were euthanized 28 days posttreatment, and their maxillae were processed for further histological analysis. The upper left first molars were used as controls for future comparisons of the tissue formation. *In vivo* procedures and treatments were performed by a single operator (endodontist), previously trained and calibrated.

#### 2.1.10. Histology

The samples were fixed into 10% buffered formalin solution, decalcified in 4% EDTA solution (pH 7.4), dehydrated, and embedded in paraffin. Tissue specimens were sectioned at 5 *μ*m and stained with hematoxylin-eosin (H&E). The neoformed tissues into the root canal were analyzed by endodontic and pathology experts that were previously calibrated.

#### 2.1.11. Immunohistochemistry

Immunohistochemistry was performed using an antibody against HSP-25 to identify odontoblast-like cells following the methodology described previously [14].

#### 2.1.12. Statistical Analysis

All experiments were repeated at least three times. Two-way analysis of variance (ANOVA), complemented by Tukey's test, was applied. The level of significance was set at *p* ≤ 0.05. Quantitative data were expressed as the mean ± standard deviation (SD).

## 3. Results

### 3.1. Chitosan Viscosity

**Figure 2:**
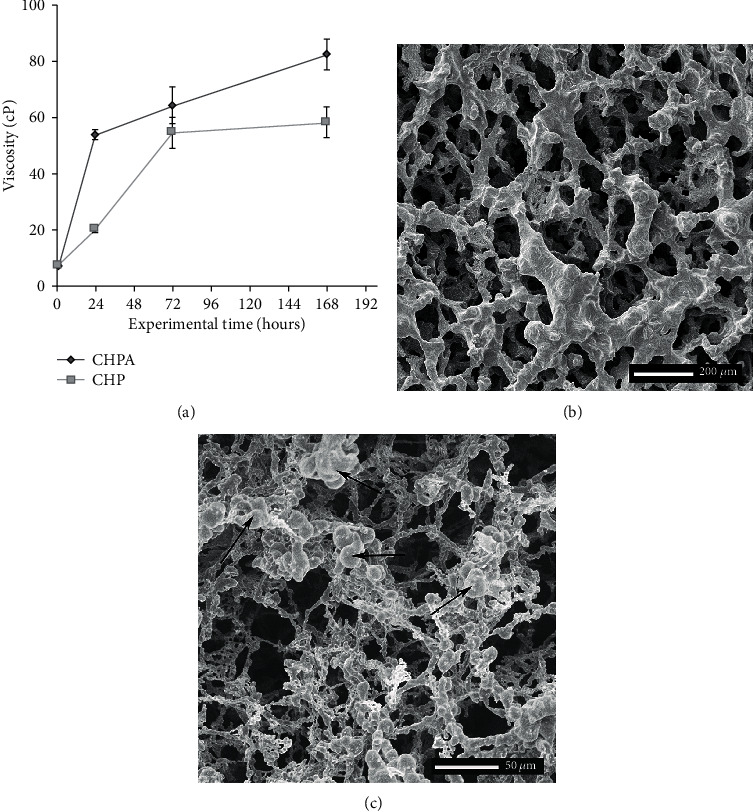
(a) Graphical representation of the viscosity of the PBS absent chitosan hydrogel (CHPA) and the chitosan hydrogel immersed into PBS (CHP) at 37°C as a function of time. (b) Illustrative scanning electron micrographs of chitosan hydrogel and (c) chitosan hydrogel seeded with SCAPs. Observe the rounded shape of the cells forming clusters of diverse sizes on top of the hydrogel beams (arrows).

The viscosity of the chitosan hydrogel increased as a function of time (Figure 2(a)). It was possible to observe an increase of more than 10-fold in the viscosity of the hydrogel kept incubated at 37°C for 168 hours (CHPA). The immersion of the hydrogel into PBS (CHP) led to a gel swelling and degradation process, with a reduction of 62% in the CHP viscosity in 24 hours and 30% after 168 hours when comparing to the CHPA.

### 3.2. Hydrogel Ultrastructure and Cell Adhesion

Illustrative scanning electron micrographs of a cross-sectional aspect of the chitosan hydrogel scaffold are presented in Figure 2(b). This ultrastructure resembles a honeycomb with pores of variable diameters interconnected to each other (Figure 2(b)). When the cells were plated inside the chitosan hydrogel, they form clusters of round cells in contact with the walls of the pores (Figure 2(c)).

### 3.3. Stem Cell Characterization

**Figure 3:**
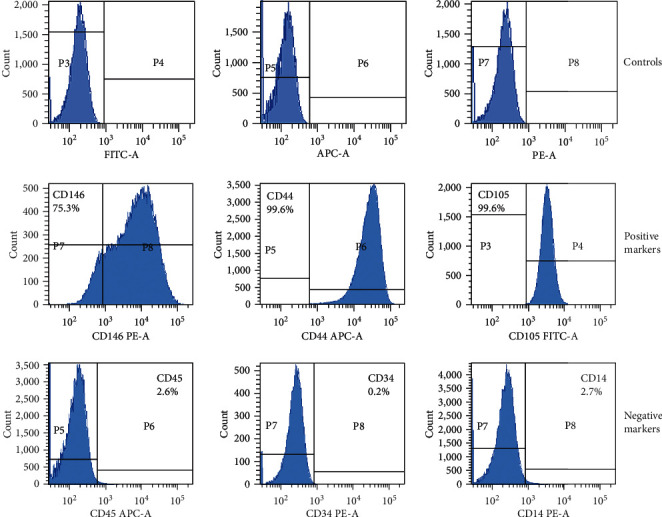
Immunoprofile of the surface molecules of the SCAPs. Positive for CD146, CD44, and CD105 and negative for CD45, CD34, and CD14, classical for mesenchymal stem cells.

SCAPs expressed levels of specific MSC surface markers, such as CD146, CD44, and CD105, and lacked the expression of the nonspecific markers of MSCs: CD45, CD34, and CD14 (Figure 3).

### 3.4. Cell Proliferation

**Figure 4:**
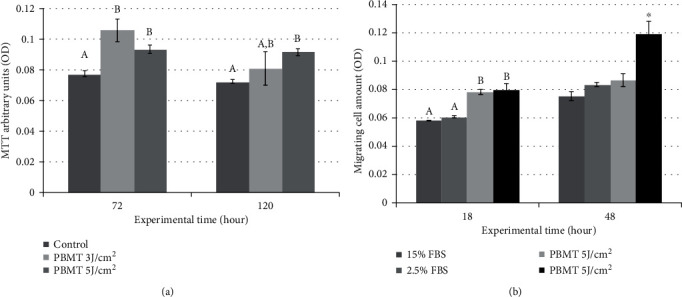
(a) Graphical representation of the number of vital cells of all experimental groups in the function of time. Different letters show statistically significant differences between groups at 72 h, whereas different capital letters show statistically significant differences between groups at 120 h (*p* < 0.05). (b) Graphical representation of the number of migrating cells of all experimental groups at 18 and 48 hours after cell seeding. Different letters show statistically significant differences between groups at the experimental time of 18 hours. ^∗^Differences between the PBMT 5 J/cm^2^ group and all other groups at 48 hours (*p* < 0.0001).

Figure 4(a) illustrates the results obtained from the MTT assay at 72 and 120 hours after cell seeding. At 72 hours, both PBMT groups showed increased cell proliferation compared to the control group. At 120 hours, only the PBMT 5 J/cm^2^ group presented a higher number of cells than the control group. At this experimental time, the PBMT 3 J/cm^2^ group presented viability similar to the control and PBMT 5 J/cm^2^ groups.

### 3.5. Migration

Figure 4(b) presents the number of migrating cells at 18 and 48 hours after seeding. At the experimental time of 18 hours, both PBMT groups (3 and 5 J/cm^2^) presented increased cell migration through the chitosan scaffold when compared to the control groups (15 and 2.5% FBS). At 48 hours, the highest migration occurred in the PBMT 5 J/cm^2^ group (*p* < 0.0001), and the all other groups presented similar migration.

### 3.6. *In Vivo* Study

**Figure 5:**
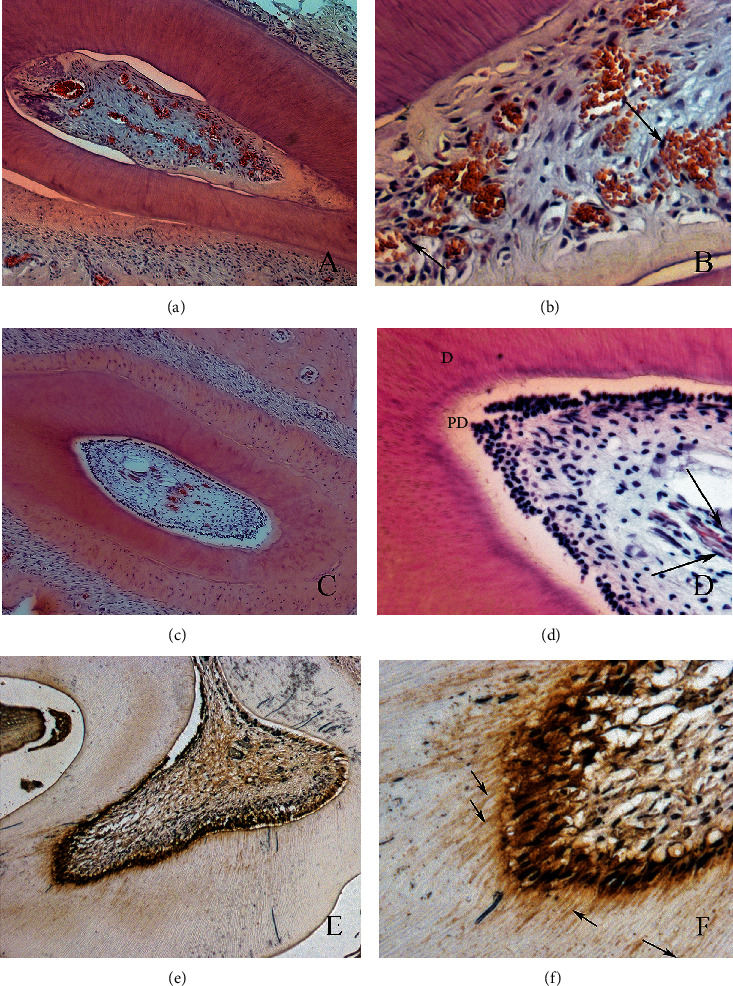
Photomicrographs illustrating the results of the blood clot+chitosan+PBMT group. Observe the newly formed connective tissue inside the root canal (a, b), with young blood vessels. This newly formed tissue is similar to healthy dental pulp (c, d). The odontoblast-like cells stained with HSP-25 (e, f) are in intimate contact with the predentin (original magnifications of (e) 10x and (f) 40x).

Two animals died at the surgery procedure. Thus, 32 teeth were retrieved for histological analysis. Representative histological sections of specimens from the *in vivo* study are presented in Figure 5. When the root canal was filled with the chitosan scaffold alone and photoactivated, it was possible to observe poor-developed tissue formation, and when it was filled with the hybrid scaffold (blood clot and chitosan), avascular tissue was observed. Well-developed pulp-like tissue with the presence of predentin along the root canal walls was observed at 4 weeks in the hybrid scaffold (blood clot+chitosan hydrogel) and PBMT group. This tissue was rich in newly formed vessels with rounded endothelial cell walls, and neither inflammation nor internal/external resorption was detected. In one tooth of this group, a layer of cells was observed in intimate contact with the dentin wall exhibiting cytoplasmic extensions penetrating the dentin tubules (Figures 5(a) and 5(b)). The healthy dental pulp tissue presented predentin and very developed and differentiated vessels with thin endothelial cell walls (Figures 5(c) and 5(d)). The newly formed tissue in the blood clot+chitosan+PBMT group was characterized by immunohistochemistry in order to identify odontoblast-like cells. Cells that were in intimate contact with the predentin lining were positive for the HSP-25 marker (Figures 5(e) and 5(f)).

## 4. Discussion

The typical treatment of pulp-compromised teeth is the root canal treatment. However, regenerative endodontic procedures, which are aimed at regenerating the dental pulp tissue, are potential alternative approaches. Two possible strategies have been explored: cell-based or cell-free (cell homing). The last one employs the host's endogenous stem cells from the apical papilla and is considered a less complex procedure and more clinically translatable. Nevertheless, the studies presented to date still point to the need for a further understanding and improvement of the cell homing strategies in order to achieve a more structured tissue morphologically and functionally similar to the dental pulp [32].

Pulp regeneration based on regenerative endodontic procedures under photobiomodulation therapy (PBMT) has shown favorable results in preclinical studies [12, 14] and might be a feasible alternative to cell-based therapies. However, the instability of the blood clot was pointed to as one of the regeneration shortcomings when a blood clot was used as a scaffold for cell homing dental pulp regeneration. To circumvent this and owing to the anatomical complexity of the root canal system, the focus on injectable hydrogel scaffolds for cell homing seems to be promising since its physical properties should favor the filling of the channels, as well as allow the migration of stem cells [33, 34].

Thus, the purpose of the present study was to evaluate the biological responses of stem cells grown inside an experimental thermosensitive and injectable hydrogel chitosan scaffold and submitted to PBMT, seeking to take advantage of the association of both approaches. The hypothesis was that the injectable chitosan hydrogel would facilitate the three-dimensional spatial organization of endogenous stem cells with the positive influence of PBMT on stem cell viability, migration, and differentiation. The biological and physical properties of this scaffold were tested *in vitro* with and without PBMT.

Chitosan in contact with dental stem cells *in vitro* promotes viability, adhesion, proliferation, and neural or odontogenic differentiation [35–39]. The chitosan hydrogel presented low viscosity due to its thermosensitive property, so it could be easily injected into the root canal. In contact with the body temperature, gelation occurs, and the viscosity is progressively increased, so it could allow the migration of the stem cells, and after that, it stabilized in a moderate viscosity with good integrity surface for regeneration. Moreover, the chitosan hydrogel presented interconnected pores, which facilitated the nutrition of stem cells inside the pores. These physical characteristics probably helped the stability of a hybrid scaffold (e.g., chitosan+blood clot) for cell homing dental pulp regeneration. In the SEM images, it was possible to notice that the stem cells used in this study (stem cells derived from the dental papilla (SCAPs)) were arranged in clusters inside the scaffold pores. Although they maintained their round shape and did not spread, they were in contact with the walls of the pores.

Photobiomodulation therapy (PBMT) was proposed by Marques et al. as “the fourth element of tissue engineering along with stem cells, scaffolds, and growth factors” [4]. This is because, due to its properties, PBMT can overcome some of the drawbacks of tissue engineering. PBMT when applied with adequate parameters modulates inflammation and improves cell migration, proliferation, and cell differentiation [5–8, 40–42], enhancing tissue formation. In the review conducted by Staffoli et al., the results showed that DPSCs (dental pulp stem cells) responded positively to laser phototherapy by improving cell growth and also suggested that PBMT may be an important tool for tissue engineering associated to stem cells [9]. In the present *in vitro* study, the influence of PBMT was observed; the SCAPs grown inside the chitosan scaffold under PBMT presented enhanced proliferation and migration, especially when the energy density of 5 J/cm^2^ was applied. These findings point out a positive response of SCAPs inside chitosan to PBMT. In fact, the possible cell sources for pulp regeneration through cell homing include dental pulp stem cells (DPSCs), stem cells from the apical papilla (SCAPs), and bone marrow stem cells (BMSCs) [43–45].

The PBMT applied with the energy density of 5 J/cm^2^ presented the most striking results for both tests, proliferation and migration. This parameter has been consistently positive in former *in vitro* studies [4, 7, 46, 47]. Marques et al. reviewed the effects of PBMT on stem cells from dental stem cells and found that the 5 J/cm^2^ energy density at 660 nm wavelength induced high rates of cell viability and proliferation. Moura-Netto et al. also reported an enhanced proliferation of stem cells from exfoliated deciduous teeth (SHEDs) under these same PBMT parameters during situations of nutritional deficit [46]. Moreover, Ferreira et al. investigated dental pulp stem cells (DPSCs) encapsulated in an injectable recombinant human Bone Morphogenetic Protein 4- (rhBMP4-) loaded hydrogel and showed that the 5 J/cm^2^ energy density improved cell survival *in vitro* and also accelerated hard tissue formation *in vivo*. In a short-term analysis, PBMT at 5 J/cm^2^ increased the number of SHEDs with no interference on their undifferentiated state [47]. In most of these studies, the PBMT was applied to cells in a stress condition. Culture medium with reduced FBS concentration causes a nutrition deficiency status with decreased cellular growth that is an appropriate condition to observe the effects of PBMT [5, 48, 49]. In the present study, this nutritional deficit approach was not necessary once the contact with the chitosan hydrogel already would represent a condition of stress. And in fact, the effects of PBMT were noticeable in the proliferation and migration analysis, mostly due to this stress condition.

Based on the *in vitro* results, we chose the energy density of 5 J/cm^2^ as the parameter to be used in the *in* vivo study. The root canals were filled with blood clot+chitosan hydrogel (hybrid scaffold), and the PBMT was carried out in contact with the crown of the tooth after the procedure. The histological analysis showed that the mesial root canal was filled with an immature connective tissue, with newly formed vessels, with morphology similar to that of healthy pulp. The immunohistochemistry identified odontoblast-like cells in intimate contact with the predentin lining. Probably, the root canal system filled with the chitosan hydrogel scaffold provided stability to the blood clot allowing the migration and adhesion of the stem cells attracted by the provoked bleeding from the root end. However, this initial colonization does not guarantee the survival of these cells over time. PBMT is a great factor with the potential to maintain cell viability, improving the proliferation and differentiation process. In addition, after stabilization, the cells start to secrete their own extracellular matrix (ECM) that could contain growth factors, which are the third element of tissue engineering and thus important for dental pulp regeneration. In this context, the PBMT also would have importance. In fact, Garrido et al. demonstrated that ECM secreted by human DPSC (dental pulp stem cell) sheets presented a higher amount of fibronectin when submitted to PBMT [41]. Also, the incorporation of fibronectin in the chitosan scaffold has been investigated and has been shown to influence the cell environment with increased adhesion and proliferation [50]. Then, in the future, the association of the chitosan scaffold with growth factors also can be tested.

In our study, PBMT was performed only after blood clot formation. Zaccara et al. tested the influence of photobiomodulation therapy on root development in rat teeth with pulp necrosis and an open apex, using cell homing and stem cell transplantation (cell-free and cell-based therapies), and the PBMT was performed on 24 h intervals for 30 days [44, 47, 50, 51]. The results showed that PBMT improved tissue response to apexification and favored the increase in root length and width (apexogenesis). They concluded that daily irradiations are important to maintain the biostimulating effect. Therefore, continuing irradiation in the days after the regenerative endodontic procedure is something that needs to be further investigated in our proposed model and can improve the results found so far.

## 5. Conclusions

The findings of the present study are promising and showed that chitosan hydrogel when applied with a blood clot as a hybrid scaffold and PBMT could in the future improve previous results of dental pulp regeneration through cell homing approaches. However, further studies need to be performed, especially those of *in vivo* cell homing-based dental pulp regeneration with long-term follow-ups.

## Data Availability

All data included in this study are available upon request by contact with the corresponding author.
